# Bioassay-Guided Isolation of DPP-4 Inhibitory Fractions from Extracts of Submerged Cultured of *Inonotus obliquus*

**DOI:** 10.3390/molecules18011150

**Published:** 2013-01-16

**Authors:** Yan Geng, Zhen-Ming Lu, Wei Huang, Hong-Yu Xu, Jin-Song Shi, Zheng-Hong Xu

**Affiliations:** 1 School of Pharmaceutical Science, Jiangnan University, Wuxi 214122, Jiangsu, China; 2 Tianjin Key Laboratory for Industrial Biological Systems and Bioprocessing Engineering, Tianjin Institute of Industrial Biotechnology, Chinese Academy of Sciences, Tianjin 300308, China

**Keywords:** *Inonotus obliquus*, DPP-4 inhibitor, bioassay-guided isolation, UPLC-Q-TOF-MS, molecular docking

## Abstract

*Inonotus obliquus* is a medicinal mushroom used in Russian and Eastern European folk medicine for the treatment of gastrointestinal cancer, cardiovascular disease and diabetes. Previous studies in our laboratory have demonstrated that the mycelium powders of *I. obliquus* possess significant antihyperglycemic effects in a mouse model of diabetic disease induced by alloxan. However, the active ingredients of mycelium powders responsible for the diabetes activity have not been identified. This study aims to identify the active ingredients of *I. obliquus* mycelium powders by a bioassay-guided fractionation approach and explore the mechanism of action of these active ingredients by using a well-established DPP-4 (an important enzyme as a new therapeutic target for diabetes) inhibitory assay model. The results showed the chloroform extract of mycelium was potential inhibitory against DPP-4. Bioactivity guided fractionation led to the identification of 19 compounds using UPLC-Q-TOF-MS. Molecular docking between the compounds and DPP-4 revealed that compounds **5**, **8**, **9**, **14**, **15** may be the active components responsible for the DPP-4 inhibitory activity.

## 1. Introduction

Diabetes is a metabolic disorder syndrome, which is caused by the interaction of multiple genetic and environmental factors. The disease can cause a series of complications such as diabetic nephropathy, neuropathy, retinopathy, macrovascular disease and coronary heart disease [1,2]. The traditional treatment with oral hypoglycemic drugs can’t completely cure diabetes and they have serious side effects such as hypoglycemia [3].

Dipeptidyl peptidase 4 (DPP-4) is a serine protease that localizes on cell surfaces and plays a major role in glucose metabolism. It is responsible for the rapid degradation of incretins such as glucagon-like peptide 1 (GLP-1) and gastric inhibitory peptide [4]. GLP-1 secreted by intestinal L cells helps control blood sugar levels since it has important physiological functions such as enhancing insulin secretion in a glucose-dependent manner, inhibiting postprandialglucagon secretion, delaying gastric emptying and stimulating growth of β-cells [5]. A new class of oral hypoglycemics called DPP-4 inhibitors work by inhibiting the action of this enzyme, thereby prolonging incretin effect *in vivo* [6]. Large numbers of experiments with DPP-4 inhibitors have shown that they are effective and safe [7,8,9,10].

*Inonotus obliquus*, commonly known as chaga mushroom, is a black parasitic fungus that grows in Nature on living trunks of Betula species. It has been used as a folk remedy in Russia, Poland and most of the Baltic countries for more than four centuries [11]. So far, many extracts from this medicinal mushroom have exhibited various biological activity such as hypoglycemic [12], anti-viral [13], antioxidant [14] and anti-tumor activities [15,16]. *I. obliquus* produce a diverse range of metabolites such as triterpenoids, steroids, polysaccharides and phenolic constituents that are responsible for its activity [17,18]. However, this fungus grows very slowly and is difficult to produce those metabolites in Nature since it is restricted to cold conditions. In addition, artificial cultivation usually takes several months and it is difficult to control the product quality. As an alternative method, growing it by submerged fermentation can provide fungal biomass of consistent quality [19].

Although *I. obliquus* can effectively treat diabetes, the mechanism of its therapeutic effect remains unclear. This study aimed to identify the active ingredients of *I. obliquus* by a bioassay-guided fractionation of a fermented product extract to search for DPP-4 inhibitory compounds. In our research, we obtained a DPP-4 inhibitory extract from the submerged culture of *I. obliquus*, identified 19 compounds in the extract by UPLC-Q-TOF-MS and we predicted their activity by molecular docking.

## 2. Results and Discussion

### 2.1. Isolation of the DPP-4 Inhibitory Components from Mycelium Powders of *I. obliquus*

**Figure 1:**
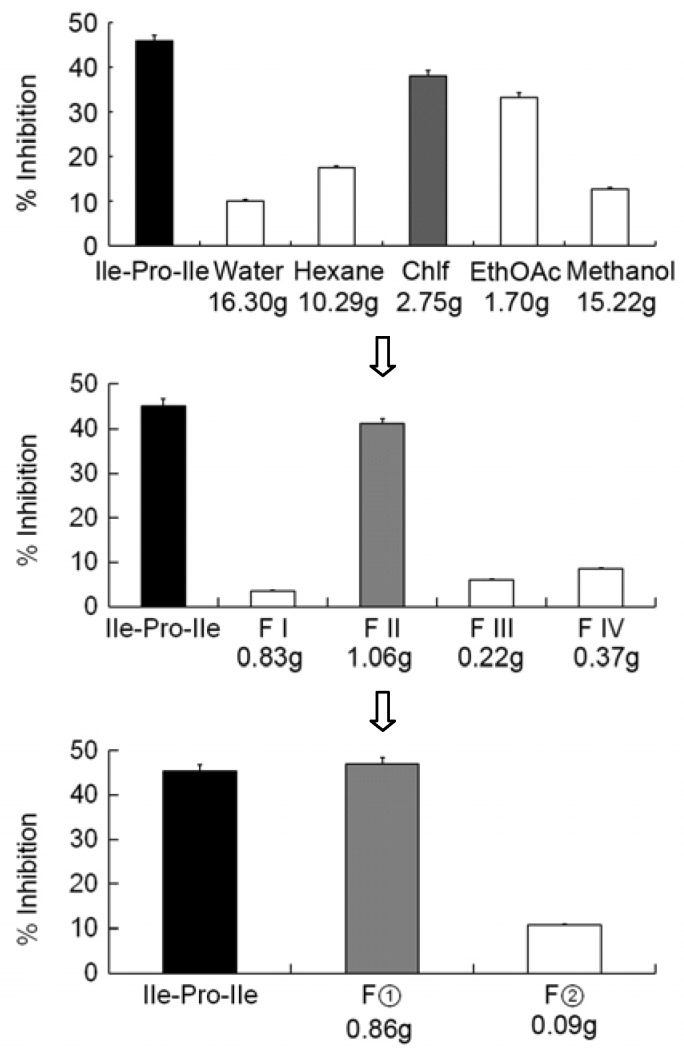
Activity-guided purification of the chloroform extract of mycelium powders. Gray represents active site. F: fraction. The results are expressed as the mean ± SD.

Figure 1 schematically depicts the extraction procedure leading to the isolation of the active compounds. One hundred and fifty grams of the *I. obliquus* mycelium powders was divided into five fractions after the extraction. The chloroform fraction (200 μg/mL) inhibited DPP-4 by 38.1%, while Ile-Pro-Ile, the positive control, had an inhibition of 45.9% (Figure 1, top). In order to characterize the active compounds within the extract, the extract was divided by silica gel column chromatography into four fractions which were then tested for DPP-4 inhibitory activity. In the four fractions, fraction II showed significantly higher inhibition of DPP-4 than the other fractions (Figure 1 middle). After the separation of Sephadex LH-20, fraction II was divided into fraction ① and fraction ②. Among these two fractions, fraction ② showed the higher activity than the positive control (Figure 1, bottom). Then fraction ② was further separated into 12 fractions using preparative HPLC.

### 2.2. Structural Determination of the Isolated Compounds

The precise identification of compounds in mixtures can be a complex task as they contain a wide variety of structures. Recently, GC-MS has been used to analyze the volatile compounds from budu (a famous Malaysian fish sauce) [20], the chemical constituents of liverwort (*Porella cordaeana*) extracts [21], and the volatile composition in brown millet, milled millet and millet bran [22]. HPLC–MS also has proved to be a very useful tool in the characterisation of natural products [23,24]. Quadrupole time-of-flight mass spectrometry (Q-TOF-MS) combines high sensitivity and mass accuracy for both precursor and product ions, providing the elemental composition of the parent and fragment ions. This feature helps to identify compounds thoroughly and to differentiate between isobaric compounds. The potential of HPLC-Q-TOF-MS for qualitative purposes has been highlighted in several studies [24,25,26,27]. 

**Figure 2:**
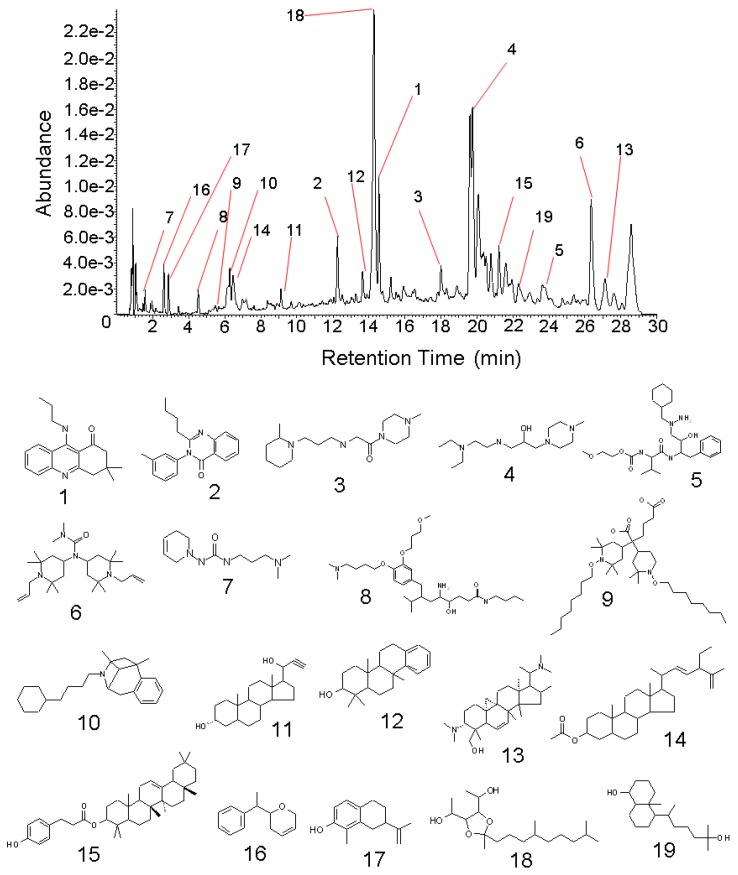
UPLC profile and compounds proposed from the active fractions.

**Figure 3:**
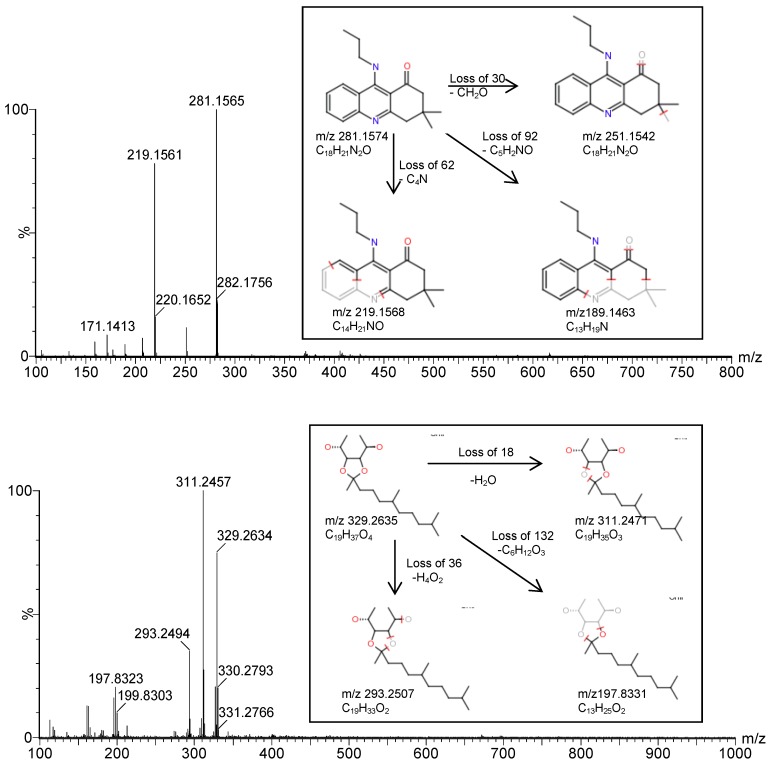
TOF MS–MS spectra and proposed fragmentation (insets) of compound 1 (above) and compound 18 (below).

Using UPLC-Q-TOF-MS, nineteen compounds were characterized from the 12 fractions. The accurate masses and molecular formulas of these nineteen compounds (Table 1) were obtained using Mass Lynx 4.1 software. The elemental composition tool also generated the number of double-bond equivalents (DBE) and i-FIT and i-FIT (Norm) (Table 1). The mass errors of the nineteen compounds were all below 5 ppm, and their i-FIT (Norm) values were low, which indicated at least 95% confidence in the accuracy of the proposed composition. To confirm the structural formulas of the nineteen compounds, we imported the potential structural formulas and their raw data file into the software Massfragment to supply the structural formulas of the fragment ions and give the fragment “soft spot”. Then the “soft spot” information and the fragmentation data of the compound supplied by the TOF-MS-MS, together with the molecular formula calculated by from the accurate mass, were used to confirm the structural formulas. Nineteen proposed compounds were identified and are shown in Figure 2. All the compounds have not been previously identified in *I. obliquus* [28,29,30,31,32]. There were ten alkaloid compounds: 3,3-dimethyl-9-(propylamino)-3,4-dihydro-1(2*H*)-acridinone (**1**), 2-butyl-3-(3-methylphenyl)-4(3*H*)-quinazolinone (**2**), 1-(4-methyl-1-piperazinyl)-2-{[3-(2-methyl-1-piperidinyl)propyl]amino}ethanone (**3**), 1-{[2-(diethylamino)ethyl]amino}-3-(4-methyl-1-piperazinyl)-2-propanol (**4**), *N*-{(1*S*,2*S*)-1-benzyl-3-[1-(cyclohexylmethyl)hydrazino]-2-hydroxypropyl}-*N*2-[(2-methoxyethoxy)carbonyl]-L-valinamide (**5**), 1,1-dimethyl-3,3-bis(2,2,6,6-tetramethyl-1-prop-2-en-1-ylpiperidin-4-yl)urea (**6**), 1-(3,6-dihydropyridin-1(2*H*)-yl)-3-[3-(dimethylamino)propyl]urea (**7**), (2*R*,4*S*,5*S*,7*S*)-5-amino-*N*-butyl-7-{4-[4-(dimethylamino)-butoxy]-3-(3-methoxypropoxy)benzyl}-4-hydroxy-2,8-dimethylnonanamide (**8**), 2,2-bis[2,2,6,6-tetramethyl-1-(octyloxy)piperidin-4-yl]-hexane-dioate (**9**) and 3-(4-cyclohexylbutyl)-6,11-dimethyl-1,2,3,4,5,6-hexahydro-2,6-methano-3-benzazocine (**10**). The remaining ten compounds were steroids, triterpenoids and other types: (3*R*,5*S*,8*R*,9*S*,10*S*, 13*S*,14*S*,17*S*)-17-(1-hydroxyprop-2-ynyl)-10,13-dimethyl-2,3,4,5,6,7,8,9,11,12,14,15,16,17-tetradeca-hydro-1*H*-cyclopent-a[a]phenanthren-3-ol (**11**), (2*S*,4a*R*,10b*R*)-1,1,4a,10b-tetramethyl-1,2,3,4,4a,4b,5, 6,10b,11,12,12a-dodecahydrochrysen-2-ol (**12**), (3β,5α,9β,16α,20S)-3,20-bis-(dimethylamino)-4-(hydroxylmethyl)-4,14-dimethyl-9,19-cyclopregn-6-en-16-ol (**13)**, (22*E*)-stigmasta-7,22,25-trien-3-yl acetate (**14**), (3β)-olean-12-en-3-yl-(4-hydroxyphenyl)propanoate (**15**), (2*S*)-2-[(1*S*)-1-phenylethyl]-3,6-dihydro-2*H*-pyran (**16**), ligudentatol (**17**), 1,6-dideoxy-3,4-O-(1,5,9-trimethyl-decylidene)-D-mannitol (**18**), (1*S*,4a*R*,5*R*,8a*S*)-5-[(1*R*)-5-hydroxy-1,5-dimethylhexyl]-4a-methyldecahydro-naphthalen-1-ol (**19**). Finally, the proposed structural formulas were elucidated by Massfragment to verify how much they matched the corresponding fragment information (two examples of matching information are shown in Figure 3).

### 2.3. Molecular Docking of the Isolated Compounds with DPP-4

Molecular docking, a key tool in structural molecular biology and computer-assisted drug design, can be used to perform virtual screening on large libraries of compounds, rank the results, and propose structural hypotheses of how the ligands inhibit the target [33]. Sitagliptin, previously identified as MK-0431 and marketed as sitagliptin phosphate under the trade name Januvia, is a well known oral antidiabetic drug of the DPP-4 inhibitor class [8]. The nineteen compounds and sitagliptin were assessed for potential fit and force field interactions with the active site of DPP-IV. The energy scores ranged from a minimum of −113.391 kJ/mol to −48.9167 kJ/mol while the energy score of sitagliptin is −90.2814 kJ/mol (Table 2). Among the nineteen compounds, compounds **5** and **8** showed energy scores of −105.071 kJ/mol and −113.391 kJ/mol which are 14.7896 kJ/mol and 23.1096 kJ/mol less than sitagliptin, respectively. There were three compounds (**9**, **14**, **15**), which are similar to sitagliptin. Table 2 showed that the root-mean-square deviation (RMSD) of compounds **5**, **14**, **15** were all small, which indicated that the conformation had changed little during the docking process. 

In traditional herbal medicine, *I. obliquus* is commonly used for treating gastritis, gastric ulcers, cardiovascular disease, several tumors and diabetes [11]. Previous studies in our laboratory have demonstrated that the dry matter of culture broth of *I. obliquus* possesses significant antihyperglycemic effect in a mouse model of diabetic disease induced by alloxan [12,34,35]. In the present study, we assessed the active anti-DPP-4 ingredients from *I. obliquus* by using the bioassay-guided fractionation approach. Ten alkaloids and nine steroids, triterpenoids and other types were identified from the active fractions of *I. obliquus*. Among these compounds, compounds **5** and **8**, showed less binding energy than sitagliptin, and were speculated to be potential inhibitors. Other compounds, such as compounds **9**, **14**, **15**, which can interact with the enzyme molecule closely, may also have inhibitory activity against DPP-4. Based on these findings, compounds **5**, **8**, **9**, **14**, **15** may be the major active ingredients of *I. obliquus* responsible for its antihyperglycemic activity. Further investigation on the structural identification and inhibitory activity in animal model of diabetes of monomer compounds collected by the preparative HPLC will be carried out in the future.

## 3. Experimental 

### 3.1. Fermentation and Product Preparation

A voucher specimen of *I. obliquus* (JNPIO01) is deposited in the Lab of Pharmaceutical Engineering, School of Pharmaceutical Science, Jiangnan University, Wuxi, China. The mycelia of *I. obliquus* were inoculated into a culture medium composed of 2.5% glucose, 0.3% tryptone, 0.15% MgSO_4_ and 0.3% KH_2_PO_4_ in distilled water with the initial pH of 4.0. Then, shaking flask culture was carried out in a 500 mL Erlenmeyer flask containing 100 mL of medium, at 25 °C for 120 h by shaking at 150 rpm/min. Hereafter, 3 L of the cultures were added to a fermenter containing 30 L fresh medium as same as above. During the fermentation, the temperature and the agitation rate are the same to shaking flask culture and the air flow rate was kept at 1.67 vvm. The fermentation was terminated at the 12 days and the mycelia were separated with broth by filtration and centrifugation. The mycelia and broth were both dried under vacuum and freeze-dried to powder form. 

### 3.2. Extraction and Isolation

One hundred and fifty grams of the fungus powders were extracted in turn with 1,000 mL hexane, chloroform, ethyl acetate and methanol three times for 12 h at room temperature and with mixing. Then, the residues were extracted with water three times for 30 min at 95 °C. After that, the samples were divided into fractions soluble in hexane (10.29 g), chloroform (2.75 g), ethyl acetate (1.69 g), methanol (15.23 g) and water (16.31 g). The chloroform-soluble part, which showed 38.1% inhibition against DPP-4 at 200 ìg/mL was fractionated on a silica gel column using a stepwise gradient of CH_3_Cl_3_-MeOH from 100:0 to 50:50. Altogether, four pooled fractions (Fractions I-IV) were collected. Fraction II (1.06g), which showed an 41.2% inhibition against DPP-4, was further separated using a Sephadex LH-20 column to give two fractions (fractions ①, ②). Fraction ① (900 mg), which showed 47.1% inhibition, was further fractionated by preparative HPLC (Waters X Bridge C18, 150 × 19 mm, 5 ìm) using a stepwise gradient of CNCH_3_-H_2_O from 40:60 to 90:10 with a flow rate of 7.2 mL/min.

### 3.3. UPLC-Q-TOF-MS Experiments

Fraction ① was analyzed by the UPLC-Q -TOF- MS hyphenated technique. UPLC was performed with a Waters (Milford, MA, USA) Acquity UPLC system equipped with a binary solvent delivery system, an autosampler and a Waters Acquity C18 column (2.1 × 100 mm, 1.7 μm). The mobile phase was a gradient prepared from acetonitrile (component A) and aqueous containing 0.2% formic acid (component B). Elution started with 10% A and the proportion of A was increased linearly to 60% in 20 min and then to 100% in 25 min. The injection volumn was 1 μL, while the flow rate was 0.3 mL/min.

Mass spectrometry was performed with a Waters Synapt Q-TOF system. Compounds were analyzed in negative ion mode. The optimized conditions were: desolvation gas 500 L/h at a temperature of 420 °C, cone gas 50 L/h, source temperature 100 °C, capillary and cone potentials 3,000 and 20 V, respectively. Data were collected in the v mode, with a scan accumulation time of 1 s and the TOF data being collected between *m/z* 100 and 1,000. The MS-MS experiments were performed using a collision energy of 25 eV. To ensure accuracy and reproducibility, all analyses were acquired using an independent reference spray via the Lock Spay interface; Tyr-Gly-Gly-Phe-Leu was used as lock mass (*m/z* 556.2771) under positive ion conditions. The Lock Spray frequency was set at 6 s, meaning that every 6 s flow from the Lock Spray was introduced into the mass spectrometer for 1 s, thus giving the software the possibility of performing ongoing correction of the exact mass of the analyte. Data for the reference compound were averaged over 10 spectra/min. The accurate mass and composition for the precursor and fragment ions were calculated using the MassLynx 4.1 software supplied with the instrument. The software has a feature that calculates all possible elemental composition from the accurate mass. When using previous knowledge, such as low i-FIT and i-FIT (Norm), type and number of atoms (*i.e.*, maximum six oxygen atoms, 17 carbon atoms, and no phosphorus), various impossible formulae can be further ruled out, this is a powerful tool for forming hypotheses about the identity of an unknown compound. Final identification can then be performed on the basis of accurate measurement of the mass of the parent ions and the fragments obtained in MS-MS experiments.

### 3.4. DPP-4 Inhibition Assay

DPP-4 activity was determined using a Chromogenic substrate method with glycine-proline *-p*-nitroaniline (Gly-Pro-PNA) as a substrate. The mixture which composed of enzyme, substrate, positive control (Ile-Pro-Ile) and Tris buffer (pH 8.2), were incubated at 37 °C for 30 min before mixing. To each well of 96-well microtiter plate was added positive control (0.29 mmol/L) or sample, DPP-4 (10 U/L), Tris buffer (pH 8.2) and substrate (4.68 mmol/L) in turn. Then the mixture was incubated at 37 °C for 2 h. The absorbance (A) of each well was measured at 405 nm with microplate reader (Thermo Scientific Multiskan Ascent). The inhibition activity was calculated by the equation:

Inhibition (%) = [(ANegative control − A control) − (A sample − A sample control)]/ (A Negative control − A control) × 100%(1)

### 3.5. Molecular Docking

A structural model was created based on the DPP- 4 crystal structure (PDB number 1pfq) using ICM-Pro and Homology (Molsoft, San Diego, CA, USA). Water molecules and irrelevant chains of the protein molecule were deleted and then the protein was converted to an ICM object with all hydrogens being optimized. To set up the docking project, we first identified potential ligand-binding sites. A ligand probe was placed to the site and a box was placed around this potential ligand-binding area. Receptor maps were generated and then ligand was docked into a rigid structure and generated 30 potential conformations. The orientation of ligand shown was the lowest energy of all the potential orientations.

### 3.6. Statistical Analysis

Data were expressed as mean ± SEM. Differences in measured variables between experimental and control group were assessed by using two-sided Student’s *t* tests. Results were considered statistically significant at *p* < 0.05.

## 4. Conclusions

In conclusion, the chloroform extract of mycelium of *I. obliquus* exhibited anit-DPP-4 activity. Nineteen compounds were characterized according to their retention times and mass spectra by UPLC-MS. Molecular docking revealed that compounds **5**, **8**, **9**, **14**, **15** may be the major active ingredients of *I. obliquus* for the treatment of diabetes, and their antihyperglycemic effect may be mediated, at least in part, by inhibition of DPP-4 activity.

## Figures and Tables

**Table 1:** Q-TOF-MS accurate mass elemental composition of the proposed compounds.

Compound	Experimental mass (*m/z*)	Theoretical mass (*m/z*)	Mass error		DBE	Molecular formula	i-FIT	i-FIT (Norm)
			mDa ppm					
1	281.1565	281.1574	−0.9	−3.2	9.5	C_18_H_21_N_2_O	150.0	0.0
2	291.1724	291.1737	−1.3	−4.4	10.5	C_19_H_19_N_2_O	46.1	0.3
3	295.2505	295.2498	0.7	2.4	3.5	C_16_H_31_N_4_O	166.9	0.0
4	271.2498	271.2498	0.0	0.0	0.5	C_14_H_31_N_4_O	79.0	0.0
5	491.3223	491.3233	−1.0	−2.0	7.5	C_26_H_43_N_4_O_5_	54.6	0.3
6	445.3879	445.3893	−1.4	−3.1	5.5	C_27_H_49_N_4_O	69.2	1.0
7	225.1876	225.1872	0.4	1.8	2.5	C_11_H_21_N_4_O	40.3	0.4
8	564.4618	564.4614	0.4	0.7	-0.5	C_32_H_57_N_3_O_5_	50.4	0.0
9	677.5576	677.5571	0.5	0.7	6.5	C_40_H_73_N_2_O_6_	36.9	0.0
10	338.2864	338.2848	1.6	4.7	7.5	C_24_H_36_N	127.2	0.0
11	329.2453	329.2467	−1.4	−4.7	6.5	C_22_H_33_O_2_	53.0	0.7
12	311.2392	311.2375	1.5	4.8	7.5	C_22_H_31_O	173.1	1.2
13	443.3663	443.3648	1.5	3.3	6.5	C_28_H_47_N_2_O_2_	100.2	0.0
14	456.3778	456.3760	1.8	3.9	7.5	C_31_H_47_O_2_	42.9	0.0
15	573.4431	573.4453	−2.2	−3.8	10.5	C_39_H_57_O_3_	36.9	0.4
16	187.1162	187.1156	0.6	3.5	6.5	C_13_H_15_O	96.8	0.4
17	201.1278	201.1279	−0.1	−0.5	6.5	C_14_H_17_O	41.4	0.3
18	329.2634	329.2635	−0.1	−0.3	1.5	C_19_H_37_O_4_	109.7	0.1
19	295.2632	295.2637	−0.5	−1.7	2.5	C_19_H_35_O_2_	121.3	0.1

**Table 2:** Docking results of the proposed compounds and DPP-4.

Compound	Molecular Weight	Energy (kJ/mol)	RMSD
**Sitagliptin**	505.3	−90.2814	1.21861
**1**	281.2	−54.5608	2.20387
**2**	291.2	−63.6279	4.57834
**3**	295.2	−67.4692	2.77483
**4**	271.2	−61.6337	6.21484
**5**	491.3	−105.071	1.47814
**6**	445.4	−64.0713	10.2671
**7**	225.2	−67.2371	6.84517
**8**	564.5	−113.391	4.66248
**9**	677.6	−86.1653	7.27826
**10**	338.3	−70.4691	3.83635
**11**	329.2	−60.2719	6.73284
**12**	311.2	−51.5762	3.88163
**13**	443.4	−61.9833	4.26127
**14**	456.4	−85.7413	1.27186
**15**	573.4	−85.1947	2.18643
**16**	187.1	−48.9167	3.37182
**17**	201.1	−52.3578	0.087938
**18**	329.3	−73.6836	8.26177
**19**	295.3	−59.8366	4.57368
